# Musculoskeletal symptoms in an adolescent athlete population: a comparative study

**DOI:** 10.1186/s12891-015-0681-4

**Published:** 2015-08-20

**Authors:** Élise P. Legault, Martin Descarreaux, Vincent Cantin

**Affiliations:** Département des sciences de l’activité physique, Université du Québec à Trois-Rivières, Trois-Rivières, G9A 5H7 Canada

## Abstract

**Background:**

Musculoskeletal pain, symptoms or injuries are prevalent in the adolescent athlete population as well as in the general adolescent population, and often have significant consequences on their future musculoskeletal health. However, differences between these two populations in regards to their musculoskeletal health are not known and have not yet been explored. Therefore, the main objectives of this study are to 1) compare the 6-month prevalence of musculoskeletal symptoms and their impact on school attendance and reduction in sport or leisure activity between a group of adolescent athletes and a group of control adolescents, and 2) determine if gender has different effects on the prevalence of musculoskeletal symptoms in these two populations.

**Methods:**

Among adolescents who participated in the 2012 Québec summer games, 1,865 agreed to participate and constituted the adolescent athletes group (mean age:14.12 ± 1.22). An additional cohort of 707 adolescents from two schools was also recruited to form the comparison control group (mean age: 14.69 ± 1.38). Anthropometric data were collected, and the musculoskeletal 6-month prevalence of symptoms and their related impacts were assessed using the Teen Nordic Musculoskeletal Screening Questionnaire (TNMQ-S). Participants’ characteristics as well as symptoms prevalence for the nine anatomical regions as well as their impact on school/work absence and reduction in physical/leisure activities were compared between athletes and control adolescents.

**Results:**

When compared to athlete adolescents, significantly more controls had a positive 6-month prevalence of symptoms affecting the neck (48.8 % vs 26.3 %), upper back (41.3 % vs 18.1 %) and low back (45.4 % vs 35.8 %) when compared to athlete. Symptoms affecting the spine led to significantly more school absence and reduction in physical activity in the control group. Controls also showed higher prevalence of shoulder (37.1 % vs 28.3 %) and wrist/hand (23.8 % vs 17.4 %) symptoms, while athletes had a higher prevalence of elbow symptoms (8.7 % vs 11.4 %).

**Conclusion:**

Despite their higher risk of injuries related to high levels of competition or sport participation, adolescent athletes have fewer symptoms affecting the spine than “typical adolescents”, and similar prevalence of symptoms affecting the body’s extremities. Further investigations are necessary to understand the differences between athletes and non-athletes in regard to disability and long-term complications associated to musculoskeletal pain or symptoms.

## Background

Musculoskeletal symptoms and injuries are prevalent in the adolescent population and often have a significant impact on their future musculoskeletal health [1–7]. Numerous studies have assessed the prevalence and incidence rates of injuries or symptoms and pain in the adolescent population. For instance, one study identified musculoskeletal pain as the second most reported physical symptom after headaches; up to 7 % of adolescents suffered from this type of symptom often or on a daily basis [5]. In 2006-2007, 380,000 adolescents and pre-adolescents from Ontario (Canada) consulted a health provider for musculoskeletal disorders, representing a consultation rate of 122 visits per 1,000 youths [4]. Furthermore, traumatic injuries (fractures, dislocations and sprains) were the most common, with a rate of 63 consultations per 1,000 youths, followed by undiagnosed musculoskeletal disorders, with a rate of 33 per 1,000 [4]. Another study found that 2/3 of the injuries sustained by Canadian adolescents aged 12–19 years occurred while practicing sports or recreational activities [6]. Clearly, traumatic injuries due to sporting or physical activities, as well as general musculoskeletal disorders, are two prevalent conditions in the adolescent population.

According to several literature reviews, higher levels of competition and training errors such as unsuitably high training volumes, or inadequate training methods are risk factors for injuries in the adolescent athlete population [1, 7, 8]. Hulsegge et al. (2011) also studied the association between physical activity and musculoskeletal complaints in a large pre-adolescent cohort [9]. The authors found that being physically active at least 5 days a week for one hour or more was associated with a significantly higher risk of lower body extremity complaints and conversely, a reduced non-significant risk of back complaints [9]. Another study from Shan et al. (2013) found that adolescents being physically active for more than 60 min, 1–4 days a week, had significantly less complaints about the lower back and the shoulders/neck [10]. On the other hand, the same study reported that adolescents who exercised for longer or shorter periods reported increased low back and shoulder/neck complaints [10]. Therefore, adolescent athletes who practice an inadequately high volume of physical activity have an increased risk of developing either injuries or symptoms, especially affecting lower body extremities, [1, 7–9, 11] and conversely, less active adolescents are more at risk of symptoms affecting the spine [10].

Additionally, gender is associated with musculoskeletal injuries, symptoms or pain in the general adolescent population [1, 9, 12]. More specifically, adolescent girls have an increased risk of injuries and symptoms or pain in specific anatomical regions such as the lower back, neck, shoulders and knees [1, 10, 13, 14]. However, it is unclear whether gender has the same effect on adolescent athletes and non-athletes, and whether this factor is associated to higher symptoms prevalence for the same anatomical regions in these two populations.

Given the association between high levels competition or training errors and injuries, adolescent athletes may have a greater prevalence of symptoms as well as more severe symptoms compared to typical adolescents. Therefore, this study has two main objectives: 1) compare the 6-month prevalence of musculoskeletal symptoms, over 9 different anatomical regions, and their impact on school attendance and reduction in sport or leisure activity between adolescent athletes and less active adolescents and; 2) determine if age and gender influences the prevalence of musculoskeletal symptoms of adolescent athletes compared to less active adolescents.

## Methods

### Study population

The Quebec Summer Games (*Jeux du Québec*) is a provincial multi-sport competition that occurs every two years and regroups more than 3,600 adolescent athletes aged between 10 and 18 years, from 19 different regions of the province of Quebec*.* All adolescent athletes who participated in the 2012 Quebec summer Games were contacted for this study. An additional sample of 1,050 students (considered a less active population of adolescents) between 12 and 17 years of age was also recruited from two different types of schools (private and public) in the region of Mauricie to form the control group. Some athletes may have been included in the control group, as it was chosen to represent a sample of typical adolescents, some being more active and others less. The adolescents and their parents were informed of the procedures and gave their written informed consent before participating in the study. This study was approved by the Université du Québec à Trois-Rivières human research ethics committee (CER-12-176-06.03).

### Data collection

The data of this cross-sectional study were collected using a questionnaire measuring socio-demographic and anthropometric information (age, gender, weight, height, region of origin and family status), the physical activity participation level, as well as the prevalence and impact of musculoskeletal symptoms. The physical activity data was collected using the short form of *The International Physical Activity Questionnaire (iPAQ)* [15]*.* The iPAQ is a valid and reliable tool used to estimate the time spent being physically active over a 7-day period [16]. Time estimates, as well as weekly frequency of physical activity were estimated and divided into three categories of intensity: 1) high intensity activities such as running, sports, or exercises that bring on significant shortness of breath; 2) moderate intensity activities such as bicycling to school and leisure physical activity that brings on light shortness of breath; and 3) walking, including walking to school or walking the dog. The symptom prevalence data was collected using *The Teen Nordic Musculoskeletal Screening Questionnaire (TNMQ-S),* which is a translated and adapted form of the *Extended Nordic Musculoskeletal Questionnaire (NMQ-E).* Validity and reliability of the TNMQ-S were assessed in another study [17]. The TNMQ-S is comprised of three dichotomous questions over 9 anatomical regions: the 6-month prevalence of musculoskeletal symptoms, the impact of these symptoms on school/work attendance, as well as their impact on sport/leisure activities. The anatomical regions included in the TNMQ-S are: the neck, the shoulders, the upper back, the elbows, the wrists/hands, the lower back, the hips/thighs, the knees and the ankles/feet. The three main questions of the TNMQ-S were formulated this way: 1) “Have you had neck symptoms (pain, ache, discomfort, throbbing) at any time during the last 6-months?”; 2)”During the last 6-months: have you missed school days or work days because of this trouble?”; 3) “During the last 6 months: have you been forced to decrease your activities (sports, leisure activities, etc.) because of this trouble?”. The purpose of the questions related to school/work absence and reduction in physical/leisure activities was to get an indication of the severity of the symptoms, which can be expressed by one or more of the following: injury type, physical activity time loss, economic cost (work absence), and clinical outcome [1].

### Procedures

Each regional team leader from the Quebec Summer Games, as well as the teachers from the two schools were briefed on the study objectives and methods for completing the questionnaire. Henceforth, they were able to adequately assist the adolescents and ensure that they completed the questionnaire individually, although some adolescents completed the questionnaire at home. Team leaders and teachers collected the completed questionnaires and transmitted them to the study researchers for analysis.

### Data analyses

Four participants from the athletes group and three participants from the control group were removed from the study’s sample since the data found in their questionnaire was clearly aberrant. Additionally, the athletes group was older and had a greater age range than the control group, which could have influenced the comparisons. Therefore, 94 questionnaires from the athletes group as well as 7 questionnaires from the control group were rejected in order to have a comparable age range between samples constituted only of adolescents aged between 12 and 17 inclusively. There was only a small amount of missing data in the musculoskeletal symptoms section, varying across the different anatomical regions from 1.4 to 1.8 % of the complete data set. These missing values did not affect the sample’s description, since no significant differences were found between the complete data set and the missing data sample concerning age, gender, height and weight.

### Statistical analysis

Statistical analyses were conducted using IBM’s SPSS Statistics® (version 21.0; SPSS Inc., Chicago, IL, USA). Participants’ characteristics as well as symptoms prevalence for the nine anatomical regions as well as their impact on school/work absence and reduction in physical/leisure activities were compared between athletes and less active adolescents using Pearson’s chi square statistics (*χ*^*2*^) for categorical variables and Mann–Whitney *U* test or *T* test for continuous variables. Crude odds ratios (95 % confidence intervals (CIs)) were calculated to compare the risk of musculoskeletal symptoms and related impacts in both groups using binomial logistic regression models. To control for gender and age between group differences data were further included in a second binomial logistic regression model to measure adjusted odds ratios (ORs) and 95 % (CIs) for risk of symptoms prevalence and their impact (athlete’s group used as reference). The level of significance was set at *p* < 0.05 for all analyses.

## Results

**Fig. 1:**
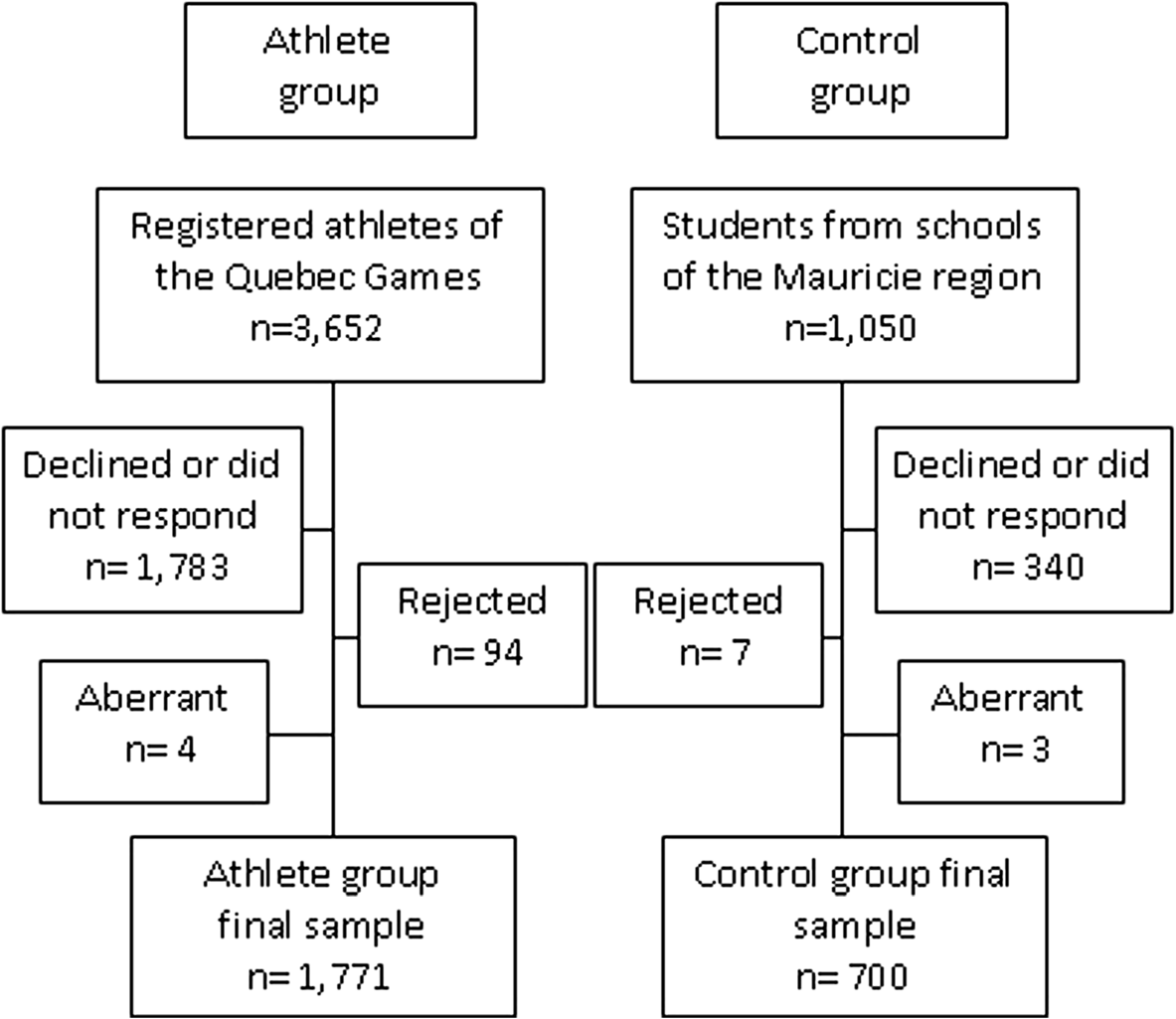
Participants’ recruitment flowchart

The response rate was 51.1 % (*n* = 1,865) for the athletes group and 67.3 % for the control group (*n* = 707). A participant flowchart (Fig. 1) depicts the recruitment procedures. After rejecting aberrant questionnaires and excluding adolescents below or above the 12 to 17 age range, the final sample size constituted of 1,771 athletes and 700 controls. Mean age for the athletes and the control groups were 14.12 ± 1.22 and 14.69 ± 1.38 respectively. A significant between-group difference (*t* = 9.481; *p* < 0.001) was observed for the mean age. There was also a significant difference in the groups gender composition (*χ*^*2*^ *=* 8.009; *p* = 0.005), as there were more male adolescents in the athletes group, representing 52.0 % of the sample compared to 45.7 % males in the control group. No significant differences were found between the groups with respect to the adolescents’ weight. However, when the age-adjusted BMI was calculated and compared, a significant difference was identified (*χ*^*2*^ = 18.759; *p* < 0.001), the control group having two times more obese adolescents compared to the athletes group. When comparing physical activity levels between groups, athletes more frequently practiced high intensity (*p* < 0.001) and moderate (*p* < 0.001) physical activity, expressed in days involving this type of activity per week, whereas controls practiced walking type activities (*p* = 0.050) more frequently. Furthermore, 59.4 % of athletes reported practicing over 420 min per week of high intensity physical activities compared to 15.4 % of controls. The sample’s descriptive data is presented in Table 1.

**Table 1:** Participant’s descriptive data

	Athlete	Control	Sig.
Sample (n)	1771	700	
Age (years)	14.12 ± 1.22	14.69 ± 1.38	*p* < 0.001
Gender n (%)			
Female	847 (48.0)	376 (54.3)	*Χ* ^2^ = 8.009
Male	918 (52.0)	316 (45.7)	*p =* 0.005
Weight (kg)	56.96 ± 11.58	57.64 ± 13.64	*t* = 1.204
			*p* = 0.229
Height (m)	1.67 ± 0.10	1.66 ± 0.10	*p* = 0.007
Age adjusted BMI n (%)			
Underweight	34 (2.1)	17 (2.8)	*Χ* ^2^ = 18.759
Healthy	1,341 (84.2)	476 (78.3)	*p <* 0.001
Overweight	154 (9.7)	65 (10.7)	
Obese	63 (4.0)	50 (8.2)	
Weekly frequency of physical activity practice (n)			
High intensity	4.90 ± 1.48	3.02 ± 1.85	*p* < 0.001
Moderate	3.79 ± 2.13	3.14 ± 2.04	*p* < 0.001
Walking	4.38 ± 2.47	4.56 ± 2.42	*p* = 0.050
Minutes of high intensity physical activity per week (%)			
0–209	323 (17.7)	388 (57.6)	*×* ^2^ = 477.263
210–419	416 (22.9)	181 (26.9)	*p* < 0.001
≥ 420	1,081 (59.4)	104 (15.4)	

**Fig. 2:**
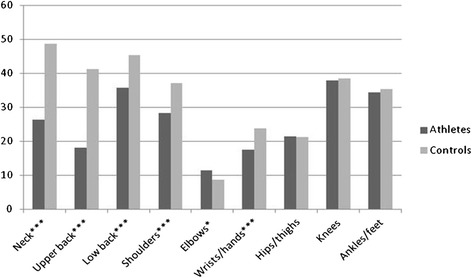
Proportion (%) of reported 6-month musculoskeletal symptom prevalence by body region. Differences between groups calculated with Pearson’s Chi-square statistic *p < 0.05; **p < 0.01; ***p < 0.001

**Fig. 3:**
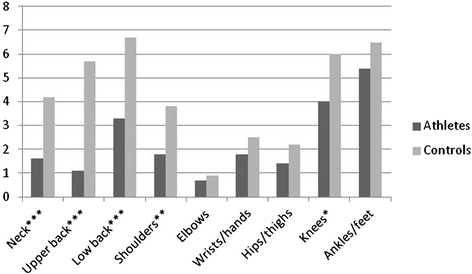
Proportion (%) of reported symptoms having caused school absence. Differences between groups calculated with Pearson’s Chi-square statistic *p < 0.05; **p < 0.01; ***p < 0.001

**Fig. 4:**
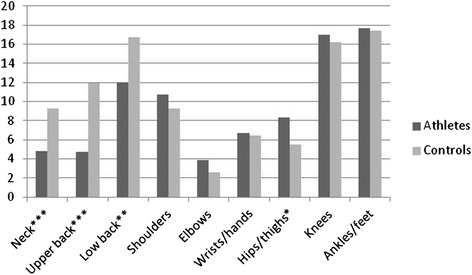
Proportion (%) of reported symptoms having caused reduction in leisure or physical activity. Differences between groups calculated with Pearson’s Chi-square statistic *p < 0.05; **p < 0.01; ***p < 0.001

The prevalence of symptoms and the related impact on school/work absence and reduction in physical/leisure activities for both groups are presented in Table 2. The data analyses (*χ*^*2*^) showed, as illustrated in Fig. 2, that significantly more controls had a positive 6-month prevalence of symptoms affecting the neck (48.8 % vs 26.3 %), upper back (41.3 % vs 18.1 %) and low back (45.4 % vs 35.8 %) when compared to athlete. These symptoms led to significantly more school absence (Fig. 3) and reduction in physical activity (Fig. 4) for the control group. Regarding the extremities, only the 6-month prevalence of shoulder, elbow and wrist/hand symptoms were found to be significantly different between groups, controls having a higher prevalence for shoulder (37.1 % vs 28.3 %) and wrist/hand (23.8 % vs 17.4 %), while athletes had a higher prevalence of elbow symptoms (8.7 % vs 11.4 %). As illustrated in Figs. 3 and 4, significantly more controls missed school because of their shoulder and knee symptoms. The hips/thighs region was the only anatomical region where athletes showed a significantly higher prevalence of symptoms having caused a reduction in physical activity.

**Table 2:** Symptom prevalence and related impact differences between athletes and controls

	Musculoskeletal symptoms prevalence 6-months			Prevalence of school and/or work absence related to symptoms			Prevalence of reduction in physical/leisure activities related to symptoms		
	Athletes n (%)	Control n (%)	*Χ* ^2^ (df) p	Athletes n (%)	Control n (%)	*Χ* ^2^ (df) p	Athletes n (%)	Control n (%)	*Χ* ^2^ (df) p
Neck	462/1754 (26.3)	335/686 (48.8)	113.444 (1)	28/1754 (1.6)	29/686 (4.2)	14.963 (1)	85/1754 (4.8)	64/685 (9.3)	17.368 (1)
			<0.001			<0.001			<0.001
Upper back	318/1754 (18.1)	284/688 (41.3)	142.566 (1)	20/1754 (1.1)	39/688 (5.7)	42.980 (1)	83/1754 (4.7)	82/687 (11.9)	40.648 (1)
			<0.001			<0.001			<0.001
Low back	628/1752 (35.8)	310/683 (45.4)	18.898 (1)	57/1753 (3.3)	46/683 (6.7)	14.728 (1)	210/1753 (12.0)	114/682 (16.7)	9.547 (1)
			<0.001			<0.001			0.002
Shoulders	496/1752 (28.3)	253/682 (37.1)	17.790 (1)	32/1752 (1.8)	26/680 (3.8)	8.392 (1)	188/1751 (10.7)	63/681 (9.3)	1.169 (1)
			<0.001			0.004			0.280
Elbows	200/1753 (11.4)	59/681 (8.7)	3.887 (1)	12/1753 (0.7)	6/679 (0.9)	0.264 (1)	68/1753 (3.9)	18/681 (2.6)	2.198 (1)
			0.049			0.607			0.138
Wrists/Hands	306/1755 (17.4)	164/690 (23.8)	12.789 (1)	31/1755 (1.8)	17/688 (2.5)	1.274 (1)	118/1755 (6.7)	44/686 (6.4)	0.076 (1)
			<0.001			0.259			0.782
Hips/thighs	377/1755 (21.5)	146/688 (21.2)	0.020 (1)	25/1754 (1.4)	15/687 (2.2)	1.760 (1)	146/1754 (8.3)	38/686 (5.5)	5.484 (1)
			0.888			0.185			0.019
Knees	665/1755 (37.9)	264/687 (38.4)	0.060 (1)	70/1753 (4.0)	41/686 (6.0)	4.466 (1)	298/1754 (17.0)	111/687 (16.2)	0.245 (1)
			0.806			0.035			0.620
Ankles/feet	600/1751 (34.3)	242/686 (35.3)	0.223 (1)	95/1751 (5.4)	44/681 (6.5)	0.976 (1)	310/1751 (17.7)	119/682 (17.4)	0.022 (1)
			0.637			0.323			0.882

Girls in the control group had a significantly higher prevalence of symptoms affecting the neck (*p* = 0.022), the lower back (*p* < 0.001), the hips/thighs (*p* = 0.014) and the knees (*p* = 0.030) than boys in the control group. Only the prevalence of elbow symptoms (*p* = 0.044) was significantly higher for boys of the control group. Athlete girls, on the other hand, had a significantly higher prevalence of symptoms than boys for nearly every anatomical region, except the elbows and the wrists/hands.

The results of the binomial logistic regressions are presented in Table 3 (Crude and adjusted odds ratios) and showed that, after adjusting for age and gender, controls had significantly lower prevalence of shoulder symptoms having caused a reduction in physical activity (OR: 0.70). For all other group comparisons, age and gender did not affect group differences.

**Table 3:** Crude and adjusted odds ratios of symptom prevalence and related impacts associated with athletic status^a^

	Musculoskeletal symptoms during the last 6-months				Impact of symptoms on school and/or work during the last 6-months				Impact of symptoms on activities during the last 6-months			
	Crude		Adjusted^b^		Crude		Adjusted^b^		Crude		Adjusted^b^	
	OR	(95 % CI)	OR	(95 % CI)	OR	(95 % CI)	OR	(95 % CI)	OR	(95 % CI)	OR	(95 % CI)
Neck	2.67	(2.22–3.21)	2.52	(2.08–3.04)	2.72	(1.61–4.61)	2.68	(1.56–4.60)	2.02	(1.44–2.83)	1.93	(1.37–2.73)
Upper back	3.17	(2.61–3.86)	2.95	(2.41–3.60)	5.21	(3.02–9.00)	4.90	(2.80–8.58)	2.73	(1.98–3.75)	2.47	(1.78–3.43)
Low back	1.49	(1.24–1.78)	1.31	(1.09–1.58)	2.15	(1.44–3.20)	1.95	(1.29–2.94)	1.47	(1.15–1.90)	1.32	(1.02–1.70)
Shoulders	1.49	(1.24–1.80)	1.27	(1.05–1.54)	2.14	(1.26–3.61)	2.06	(1.20–3.52)	0.85	(0.63–1.14)	0.70	(0.51–0.96)
Elbows	0.74	(0.54–1.00)	0.68	(0.50–0.93)	1.29	(0.48–3.46)	1.24	(0.45–3.41)	0.67	(0.40–1.14)	0.61	(0.35–1.04)
Wrists/Hands	1.48	(1.19–1.83)	1.36	(1.09–1.69)	1.41	(0.77–2.56)	1.42	(0.77–2.61)	0.95	(0.66–1.36)	0.86	(0.59–1.24)
Hips/thighs	0.98	(0.79–1.22)	0.99	(0.79–1.24)	1.54	(0.81–2.95)	1.59	(0.82–3.08)	0.65	(0.45–0.93)	0.67	(0.46–0.97)
Knees	1.02	(0.85–1.23)	1.04	(0.86–1.25)	1.53	(1.03–2.27)	1.63	(1.08–2.45)	0.94	(0.74–1.19)	0.94	(0.74–1.21)
Ankles/feet	1.05	(0.87–1.26)	1.09	(0.90–1.32)	1.20	(0.83–1.74)	1.23	(0.86–1.85)	0.98	(0.78–1.24)	1.02	(0.80–1.29)

^a^Athletes are used as the reference group

^b^Adjusted for gender and age

## Discussion

The present study shows that symptoms affecting the spine are frequent in the general adolescent population. On the other hand, lower extremities and lower back symptoms, are common conditions in the athletic adolescent population. Controls were found to have a significantly higher prevalence of symptoms affecting the spinal regions including the neck, the upper back and the lower back. When assessing the upper and lower extremities, the adolescent athletes’ symptom prevalence did not significantly exceed the prevalence observed in the control group except for the elbow symptoms. In fact, the controls had a significantly higher prevalence of shoulder and wrist/hand symptoms. Age and gender did not play a significant role in the relationship between musculoskeletal symptoms prevalence/impacts and the adolescent status (athletes vs controls).

The current results regarding symptom prevalence rates for specific anatomical regions are similar to previous findings for both adolescent athletes and controls. For instance, neck symptoms are the most prevalent musculoskeletal symptoms in the control cohort, which is consistent with the findings of other studies conducted in the general adolescent population [13, 18, 19]. These studies identified non-specific musculoskeletal pain affecting the neck and the lower extremities as the most common painful condition in the general adolescent population [13, 18, 19]. More specifically, El Metwally et al. (2004) reported prevalence of 45.4 % and 52.8 % for neck and lower limb pain respectively in their adolescent cohort over a 3-month recall period [19]. However, unlike the method used in the present study, these authors did not differentiate the lower extremities into three distinct categories. Therefore, in order to allow comparisons, the categories hips/thighs, knees and ankles/feet were regrouped, which resulted in a prevalence rate of symptoms affecting at least one of these three lower extremities of 60.9 %. This is consistent with the findings of the studies previously described. Low back pain is also a prevalent complaint in the general population, with rates reaching 20 to 50 % during adolescence [20–22]. The current study reports similar results, with a prevalence of 45.1 % for low back symptoms in the non-athletic population. Furthermore, multisport studies and reviews show that ankles and knees are the most injured body regions in active or athletic adolescent populations [1, 6, 23, 24], which is consistent with the highest prevalence rates by anatomical region found in the adolescent athletes group of this study. Low back symptoms are also prevalent in the present athlete’s cohort, though slightly inferior to the prevalence of pain identified in another study. Schmidt et al. (2013) found a 12-month low back pain prevalence of 56.0 % in their cohort of athletes, compared the current study’s low back symptom prevalence of 35.8 %. This difference could be attributed to the longer recall period (12-month) used in their study, the fact that their athlete cohort was selected from a Center for Orthopaedics and Traumatology, and the fact that they were from different sporting disciplines than those practiced by the present study’s athletes. Future studies should investigate the impact of sports related back pain during adolescence on long term disability and pain recurrences.

Caution must be applied when comparing injuries and symptoms or pain rates, since these conditions are different, but not mutually exclusive. For instance, Caine et al. (2006) found multiple definitions for injury in their review, such as new symptoms or complaints, decreased function of a body part or decreased athletic performance, cessation of practice or competition activities, and heath professional consultation. Symptoms in the current study were described as ache, pain or discomfort. The term symptom was chosen for the present study since it is more general and facilitates the comparison between adolescent athletes and non-athletes.

As mentioned before, the study results show that adolescents in the control group had a significantly higher prevalence of symptoms affecting the spine, the shoulders and the wrists/hands than athletes. Similar results were found in a study aimed at assessing differences between adolescent athletes and controls relative to their health-related quality of life as measured by two validated questionnaires [25]. In their study, Snyder et al. (2010) found that adolescents in the control group had significantly higher bodily pain scores, while the athletes scored higher for the sport and physical functioning subscale, the general health perception score, as well as happiness score [25]. Since the control group’s adolescents of the present study were found to be significantly less active than athletes, lack of physical activity may be one reason explaining these results. In fact, Shan et al. (2013) found that adolescents being active for less than 60 min, 5 days a week, had significantly more low back and shoulder/neck complaints [10]. However, another study found no significant associations between back pain and levels of physical activity as measured by accelerometer [26]. Similarly, Auvinen et al. (2008), reported that inactive adolescents did not significantly have more low back pain than their moderately active peers [27]. There seems to be divergent results regarding the association between low back pain and physical activity. Therefore, the higher prevalence of symptoms affecting the lower back found in this study’s controls may or may not be explained by their level of physical activity.

According to multiple studies and reviews, gender has a significant impact on both musculoskeletal symptoms/pain and injury prevalence or incidence in the adolescent population, especially with regard to specific anatomical sites [1, 7, 10, 13, 14]. For instance, adolescent girls have significantly more neck, upper back, shoulder and low back pain than boys, which partially concurs with this study’s findings [10, 13, 14]. In fact, control adolescent girls in this study’s control group had a significantly higher prevalence of neck, low back, hips/thighs and knees symptoms compared to boys from the same group. Results found in the athletes group were dissimilar, since gender differences were found for 8 of the 9 anatomical sites, females being more symptomatic for every region with the exception of the elbows, where males were more symptomatic, and the wrists/hands, for which no gender difference could be observed. Studies show that female athletes are more susceptible to injury than males in various sport disciplines, which is also consistent with this study’s findings [1, 7]. Finally, athletic status does not seem to strongly influence the association between musculoskeletal symptoms and gender.

To our knowledge, no other study compared symptoms from 9 different anatomical regions between adolescent athletes and less active adolescents. The survey of musculoskeletal symptoms in the adolescent population either related to a sport injury or not, is an important component in detecting and preventing musculoskeletal injuries or pain and their related consequences. Furthermore, the comparisons by athletic status or by gender may offer a better understanding of these different populations musculoskeletal health, thus making it easier to identify specific issues that need more investigation. The fact that adolescent girls have a higher prevalence of symptoms than boys and that this difference is accentuated by the athletic status of the adolescent is an example of an issue needing more investigation. Since training methods are a risk factor for musculoskeletal pain or injury, a better adaptation of training methods to better suit adolescent girl may help reducing symptom or pain prevalence in this specific population. Additional research is necessary to properly identify the factors explaining the gender differences between athletes and less active adolescent.

Athletes are known to be at higher risk of musculoskeletal injuries, especially those affecting the lower extremities [1, 23]. However, it was not clear whether this specific population would be more symptomatic than the average adolescents. The results of this study suggest that adolescent athletes who practice high levels of physical activity do not have a higher prevalence of musculoskeletal symptoms than less active adolescents. Moreover, these symptoms have similar impacts on school/work absence and physical/leisure activity loss, than those of the average adolescent. Health benefits related to a physically active life style during adolescence are numerous, such as increased self-esteem, better social skills, improved body composition, higher bone mineral density, better cardiovascular and musculoskeletal fitness, improved cholesterol levels and blood pressure [28, 29]. Given the current findings, health related benefits may outweigh the musculoskeletal risks of symptoms or injury related to a physically active life style. However, some injuries can cause long-term or permanent damage to the growing adolescent body and must therefore be taken seriously [30]. The severity and the long-term consequences of symptoms, due to injury or not, being beyond this studies scope, future studies should investigate the differences between athletes and non-athletes in regard to disability and long-term complications associated with their respective pain or symptoms. Furthermore, girls from the athletes group having higher musculoskeletal symptoms than boys, but not necessarily higher prevalence than the less active adolescent girls, future studies should focus on the development of sport programmes that are specifically tailored for adolescent girls in an effort to prevent injury occurrence and sport participation dropout in this specific population.

### Limitations

Since the response rate was 51.1 and 67.6 % for the athletes and control groups respectively, the samples may not be representative of the adolescent athlete population. Moreover, although instructions on how and when the questionnaires should be distributed to the athletes were standardized across the regional and school leaders, indications to participants on how to complete the questionnaire may have varied across groups of athletes and adolescent controls. The socioeconomic status, not evaluated in the present study, may be considered as a potential confounder in the assessment of adolescent musculoskeletal health. However, as described by Mcbeth et al. (2007), the association between socioeconomic factors and adolescent musculoskeletal health remains unclear. Assessment by self-reported questionnaires has certain limitation such as response bias caused by either acquiescence, socially desirable responding, or extreme responding [31, 32]. Acquiescence, or yessaying is defined as the tendency to answer positively to a question based on only the minimal amount of evidence, or alternately the tendency to answer too conservatively and answering positively only if the experience that is inquired has happened regularly [32]. This type of response bias could lead to either an overestimation or an underestimation of a problem. Also, as stated in a review by Shephard et al. (2003), responses from self-reported questionnaire can often be influenced by social desirability, resulting in overestimated physical activity levels and underestimated sedentary activities such as time spent watching television [31]. However, this social desirability bias only has a slight impact on this study’s results since the physical activity data was only used to properly demonstrate that the group of athletes was significantly more active than the control group.

Another study limitation is the lack of precision regarding the severity of outcome measures, which do not account for the long-term effects of symptoms and cannot distinguish between chronic or isolated symptom occurrences. Therefore, it is not possible to confirm or infirm that adolescent athletes have a better long-term musculoskeletal health than their less active peers. Finally, as mentioned earlier, some athletes may have been included in the control group, as it was chosen to represent a sample of typical adolescents, which also constitutes a limit.

## Conclusion

According to the results of the present study, less active adolescents have a higher 6-month prevalence of symptoms affecting the spine, the shoulders and the wrists/hands than their athletic peers. Furthermore, athletes have fewer symptoms affecting the spine than less active adolescents and do not have a higher prevalence of symptoms affecting the lower or upper extremities, except for the elbow. Adolescent athletic girls also have a significantly higher prevalence of symptoms for nearly every anatomical region compared to athletic boys, but do not have a higher prevalence of symptoms than less active adolescent girls. Whether or not adolescent athletes have more long-term complications or significant disability following episodes of musculoskeletal symptoms, compared to non-athletes, remains to be investigated and future studies should be oriented towards the prevention of sport dropout or the development of structured and accessible sport and physical activity programs that limit injury occurrence.

## Abbreviations

CIs: Confidence intervals
iPAQ: The international physical activity questionnaire
n: number
m: meter
df: degrees of freedom
NMQ-E: Extended Nordic musculoskeletal questionnaire
ORs: Odds ratios (ORs)
*χ*^2^: Pearson’s chi square statistics
TNMQ-S: Teen Nordic musculoskeletal screening questionnaire
